# Genome-wide linkage and association mapping of disease genes with the GAW14 simulated datasets

**DOI:** 10.1186/1471-2156-6-S1-S41

**Published:** 2005-12-30

**Authors:** Kim W Carter, Pamela A McCaskie, Lyle J Palmer

**Affiliations:** 1 Laboratory for Genetic Epidemiology, Western Australian Institute for Medical Research, UWA Centre for Medical Research, University of Western Australia, Ground Floor, B Block, Hospital Avenue, Nedlands, Western Australia

## Abstract

We combined the results of whole-genome linkage and association analyses to determine which markers were most strongly associated with Kofendrerd Personality Disorder. Using replicate 1 from the Genetic Analysis Workshop 14 Aipotu, Karangar, Danacaa, and New York City simulated populations, we determined that several markers showed significant linkage and association with disease status. We used both SNP and microsatellite markers to determine patterns and chromosomal regions of markers. Three consistently associated markers were C01R0050, C03R0280, and C10R0882. Using generalized linear mixed models, we modelled the effect of the three predefined phenotypic categories on disease status and concluded that the phenotypes defining the "anxiety-related" category best predicted the outcome.

## Background

Whole-genome linkage analyses involve looking for coinheritance of chromosomal regions with disease in families. Association studies seek to determine differences in the frequency of genetic variants between individuals exhibiting or not exhibiting a phenotype of interest (commonly case-control status). Family-based association studies utilize the available pedigree genetic variations to determine whether the transmission of particular genetic variants is associated with disease status. The results of linkage and association studies have been successfully combined in many analyses to refine the location of disease genes and to test the involvement of candidate genes in disease. The aim of this contribution was to perform linkage analyses, in combination with association analyses, on replicate 1 of the simulated Genetic Analysis Workshop 14 (GAW14) data to determine which markers or regions of markers are associated with Kofendrerd Personality Disorder (KPD).

## Methods

### Recoding the data

The GAW14 problem 2 description states because of the "varied phenotypes" for KPD, the "nosology for KPD falls into three different classifications", and that all three are used in diagnosis. The three main groups of phenotypes are indicative of three different methods used by each population for disease ascertainment. The different ascertainment methods and phenotypic categories suggest that complex interactions may be a key factor in identifying the causes and genetic determinants of KPD. Because we were blind to the simulated dataset answers, we chose to recode the data into these three additional grouped phenotypes to determine if complex combinations of phenotypes are of importance, in addition to examining the relationship between individual phenotypes and affection status. We chose replicate 1 as a representative data set for each of the four simulated populations. The first category, consisting of phenotypes *a *through *e*, is referred to as "communally shared emotions" (CSE). This was constructed in the data by assigning a positive affection status to an individual if they possessed at least one of phenotypes *a *through *e*, and assigning an individual a negative affection status otherwise. This procedure was similarly performed for the second category, consisting of phenotypes *f *through *i*, termed "behavioral-related" (BR) and for the third category, comprising phenotypes *j *through *l*, referred to as "anxiety related" (AR). This recoding procedure allowed us to assess affection status not only in terms of an overall status, but also in terms of the three different methods adopted by the different populations for deciding disease ascertainment.

### Linkage analysis

To perform linkage analysis on the simulated datasets, we used the MERLIN [1] pedigree analysis software package. We performed a nonparametric linkage analysis using primary affection status and CSE, BR, and AR as binary outcomes. JLGRAPH [2] was used to generate linkage graphs for each chromosome for each population from the MERLIN results.

### Association analysis

To perform association analysis on the binary traits for the simulated datasets, we used the computer program QTDT [3] to perform family-based tests. We performed an association analysis using affected individuals, including producing empirical *p*-values. The QTDT result files were input to JLGRAPH to produce association graphs for each chromosome for each population.

### Regions of interest

The results from the linkage and association analyses were collated to provide a list of potential regions of interest for further study. Each of the 917 SNPs and 416 microsatellites markers were examined to determine their significance (in terms of both linkage and association) for affection, CSE, BR, and AR, for each of the four populations. Marker regions that appeared to be significant for both linkage and association were closely examined. Candidate packets of markers consisting of potentially important SNPs were "purchased" in order to analyze chromosomal regions of interest in fine detail. The procedures outlined above for association and linkage analysis were then repeated, this time incorporating the new marker sets.

To determine the effectiveness of each of the three newly defined categories for disease ascertainment, the data were modelled using a generalized linear mixed model (GLMM). Affection status was used as a binary outcome, with CSE, BR, and AR as factored predictor variables and family ID as a clustering variable. A mixed effects model was used so that a random intercept could be fitted, defining individuals within families to be correlated.

## Results

### Linkage and association

Using the compiled list of markers from the results of the linkage and association analyses, we produced chromosomal graphs for each population in terms of SNP markers and microsatellite markers. From these initial graphs, we proceeded to select regions that appeared to be of most significance for linkage and/or association. We ranked the markers by *p*-value to determine those of highest significance. Table 1 shows nine SNP markers we determined to be significant with respect to linkage and association across all of the populations (*p*-values for each marker represent the most significant score from affection status, CSE, BR, and AR).

After "purchasing" more packets of markers and analyzing these in conjunction with the marker information already provided, several chromosomal regions showed significant linkage and association with disease status. The additional fine mapped packets available for download contained mostly SNP markers and we consequently determined that SNP markers would be of higher importance than microsatellites. In particular, regions surrounding the SNP markers C01R0050, C03R0280, and C10R0882 were examined. We describe the region surrounding marker C03R0280 as follows.

**Figure 1:**
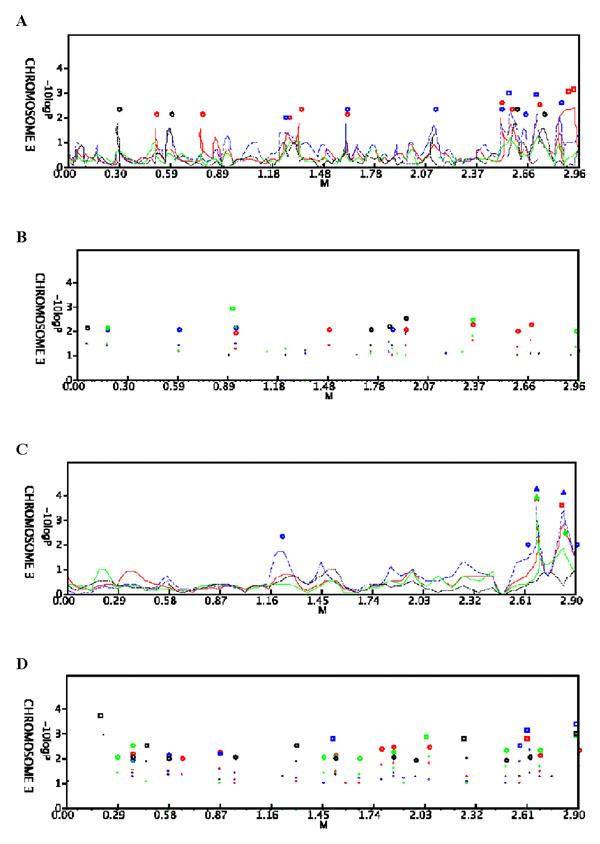
**Aipotu SNP and microsatellite linkage and association on chromosome 3. **A circle indicates the marker has a *p*-value < 0.05, a square indicates *p*-values < 0.01, a triangle indicates *p*-values < 0.001. Affection status is shown in red, with categories CSE, BR, and AR shown in blue, green, and black, respectively. A, SNP linkage: B, SNP association; C, microsatellite linkage; D, microsatellite association.

SNP marker C03R0280 is located 2.94 M along chromosome 3 of the simulated population. Figure (1A and 1B) shows linkage and association patterns using SNP markers for chromosome 3 within the Aipotu population. The region around marker C03R0280 shows significant results in terms of both the linkage and association using SNPs from the Aipotu population. Significance is visualized in the graphs by small *p*-values (indicated by large values on a -log_10 _scale) forming peaks over relevant markers. Figure (1C and 1D) represents results from the same population and chromosome for linkage and association analysis respectively, using microsatellite data. The region corresponding to the location of C03R0280 shows similar significance for both linkage and association. The finer points (circles) in Figure 1B and 1D are the actual datapoints for -log_10_P, while the larger shapes above them are used to highlight datapoints with significant *p*-values. The chromosome 3 region from approximately 2.5 M to 2.9 M shows a number of significant *p*-values for markers in terms of linkage and association for both SNP markers and microsatellites. Other chromosomes, such as 8 (not shown), provided very little evidence for linkage or association with KPD across all of the populations.

### Disease ascertainment variables as predictors of disease

Phenotypic data were modelled using GLMMs to determine how effective each of the three categories used for disease ascertainment were at predicting overall affection status for each population. For all four populations in the simulated data, AR was the only category that effectively predicted disease status (*p*-value < 0.05).

## Discussion

Table 1 highlights linkage and association *p*-values for SNP markers across the ten chromosomes. While both markers from chromosome 1 had the most significant *p*-values, closer examination of these reveals our reason for choosing chromosome 3. The two chromosome 1 markers from Table 1 were localized to linkage with affection status in the New York City population only. In comparison, marker C03R0280 showed significant linkage in three of the four populations, and C03R0199 in two populations (results not shown). Because the individual *p*-values for chromosome 1 were not replicated across more than one population, we examined chromosome 3, in particular marker C03R0280, in more detail.

The microsatellite datapoints in Figure 1C and 1D were more sparsely located, as there were lower total numbers of markers, but overall trend pattern followed those of the SNP markers. While Table 1 showed only highlights from SNP markers, chromosomal regions such as from 2.5 M to 2.9 M in chromosome 3 returned significant results for both linkage and association for both SNP and microsatellite markers (other chromosomes not shown).

By defining the three new phenotypic categories we have created an effective method for determining the particular category (or categories) that contributed to significant linkage and association for affection status within markers for each population. This flexibility enabled us to locate markers such as C03R0280 and determine that this particular marker had significant *p*-values for linkage across all populations for affection status and two of the three additional categories. Creating phenotype categories allowed us to examine groups of phenotypic effects, and to determine the contribution of subsets to the overall disease status. This approach can provide very valuable information for conducting further analysis by narrowing down target phenotypes or phenotypic groups.

## Conclusion

Through linkage and association analyses, markers C01R0050, C03R0280, and C10R0882 and markers surrounding these were found to be associated with affection status and the three phenotypic categories we defined (to varying degree within each region). Given time and "budgetary" constraints imposed on GAW14 participants, we successfully identified three gene regions, one within each of the three regions examined. While we did not find all the disease-associated genes contained within the GAW14 simulated datasets, we were successful in locating genes in the regions we focused on, indicating that our linkage and association mapping approach can successfully identify genes.

## Abbreviations

AR: Anxiety related phenotypes *j-l*

BR: Behavior related phenotypes *f-i*

CSE: Communally shared emotions phenotype *a-e*

GAW: Genetic Analysis Workshop

GLMM: Generalized linear mixed model

KPD: Kofendrerd Personality Disorder

SNP: Single-nucleotide polymorphism

## Authors' contributions

KWC performed the linkage and association analysis, generated the graphs and drafted the manuscript. PAM designed the GLMM framework, conducted the data modeling, and drafted the manuscript. LJP conceived of the study and participated in the design and coordination of the study.

## Figures and Tables

**Table 1:** Markers determined to be the most significant from linkage and association analysis

Marker	Physical location	Linkage *p*-value	Association *p*-value
C01R0050	Chromosome 1 0.52 M	0.0006	0.0009
C01R0074	Chromosome 1 2.40 M	0.0020	NA^a^
C03R0199	Chromosome 3 0.35 M	0.0030	0.0094
C03R0280	Chromosome 3 2.94 M	0.0030	0.0399
C05R0379	Chromosome 5 0.20 M	0.0140	NA
C07R0589	Chromosome 7 0.54 M	0.0009	0.0483
C07R0640	Chromosome 7 2.12 M	0.0002	NA
C09R0785	Chromosome 9 0.70 M	0.0100	0.0276
C10R0882	Chromosome 10 0.75 M	0.0300	NA

^a^NA, a marker showed strong linkage, but not association.
